# Board game on sexually transmitted infections for imprisoned women

**DOI:** 10.1186/s12905-023-02801-6

**Published:** 2024-01-05

**Authors:** Isaiane da Silva Carvalho, Ryanne Carolynne Marques Gomes Mendes, Laís Helena de Souza Soares Lima, Gabrielle Pessôa da Silva, Monique de Freitas Gonçalves Lima, Tatiane Gomes Guedes, Francisca Márcia Pereira Linhares

**Affiliations:** Graduate Program in Nursing, Federal University of Pernambuco, Recife, PE Brazil

**Keywords:** Health education, Sexually transmitted diseases, Women, Prisons

## Abstract

**Introduction:**

The board games is an educational technology that represents an appealing, active and playful pedagogical strategy and may be capable of motivating imprisoned women to learn about Sexually Transmitted Infections.

**Methods:**

A methodological study to develop and evaluate a board game, following these stages: 1. Integrative literature review to identify educational technologies on Sexually Transmitted Infections used by imprisoned women; 2. Development of the board game; and 3. Content validation performed by 23 evaluators and semantic evaluation carried out with 10 imprisoned women who were enrolled in a school located within a female prison unit in the city of Recife, state of Pernambuco, Brazil.

**Results:**

The board game consisted of the following: 01 board; 01 instructions manual; 05 pawns; 52 cards; and 01 dice. A global Content Validity Index of 0.966 was reached in the content validation process performed by health and education professionals. In the validation of the board game content regarding appearance, performed by designers/developers, most of the items obtained a Content Validity Coefficient below 0.85, which resulted in the need for adjustments and a new validation round with these professionals, in which Content Validity Coefficient = 0.917 was obtained. In the semantic evaluation, all the women stated that they improved their knowledge, increased their motivation to attend the class and would like to play the board game again.

**Conclusions:**

The “Previna” board game has been validated and can be considered an important pedagogical tool in the construction of knowledge in relation to the prevention, treatment and control of Sexually Transmitted Infections in the female prison context. The quality of this educational technology is directly related to its development based on an appropriate theoretical and methodological framework, in addition to satisfactory feedback from the target audience.

**Trial registration:**

Not applicable.

## Introduction

Imprisonment exerts a negative effect on women’s health and well-being, making them more vulnerable to Sexually Transmitted Infections (STIs) [1]. This vulnerability can be associated with lack of knowledge about preventive measures, sexual violence, risky sexual behavior, drug use, irregular or infrequent condom use of condoms, and restricted access to health services and professionals [2, 3].

The imprisoned women’s knowledge about STIs assists in the prevention and control of these infections, as it can allow change in behavior, self-care, control of risk factors and adherence to the treatment. Knowledge acquisition can occur through the implementation of health education actions in the prison environment with an emphasis on methodologies that stimulate the teaching and learning process [4].

However, lack of access to the diverse information and educational activities related to transmissibility and prevention of STIs increases women’s exposure to risk behaviors, which consequently increases the prevalence of these infections in the prison environment [5].

Health education is fundamental to avoid risks to the sexual and reproductive health of imprisoned women when carried out, above all, through educational interventions and technologies, as these play an important role in STI prevention, treatment, diagnosis or control [6, 7].

Educational technologies are used as facilitators of the teaching and learning process, are employed in the health area as a means to disseminate information and knowledge, and can provide people with active and dynamic learning, capable of causing changes and favoring actions that influence the health and disease process [8]. These technologies can present varied modalities, classified as tactile and auditory, expository and dialogical, or printed and audio-visual [9].

Games stand out among them, especially board games, which have been applied as an educational technology aimed at children, adolescents and older adults and with varied themes related to oral health, viral diseases (dengue), cardiovascular health, breastfeeding and HIV/AIDS [10–14]. This type of game emerges as an appealing, active and playful pedagogical strategy that can be used in various knowledge fields [15], and may be capable of motivating imprisoned women to learn about STIs.

This becomes necessary due to the consequences of the lack of knowledge about STIs presented by this population, as shown in a Brazilian study that demonstrated that not having knowledge about AIDS doubled the chance of acquiring the infection (OR = 2.84; 95% CI 1. 16–6.79) when compared to those who knew the disease. Furthermore, lack of knowledge about condoms increased the chances of having an STI (OR = 3.65; 95%CI 1.16–7.97) [2]. In this study, a board game called “Previna” was created and validated in terms of content and evaluated in terms of semantics, in order to assist in the construction of knowledge about STI prevention, treatment and control in the female prison environment. The knowledge by itself is known to be not enough to change behaviors, but this game can contribute to increasing incarcerated women’s knowledge about STIs and help them make the best possible decisions in terms of sexual health, with a focus on risk reduction.

In addition, the increase in the prevalence and consequences of STIs in the female prison context show the need to carry out educational actions that contribute to the quality of imprisoned women’s sexual life. Consequently, the objective of this study is to develop and validate a board game on STIs for imprisoned women.

## Methods

### Design

This is a methodological study. The research was approved by the Research Ethics Committee of the Federal University of Pernambuco (CAAE: 30035520.7.0000.5208). The process to develop the educational technology in question involved the following stages: 1. Conduction of an integrative literature review to identify educational technologies on Sexually Transmitted Infections used by imprisoned women; 2. Development of the educational technology in question guided by a methodological framework that is coherent with the technology chosen; and 3. Content validation in charge of expert judges and semantic evaluation by the target population.

### Participants

A total of 23 evaluators [16] took part in the content validation stage, of which 13 mastered content in the areas of sexual and reproductive health and/or women’s health, 05 were from the education area and another 05 from the game design/development area. The inclusion criteria adopted were based on the expert classification system adapted from the Fehring model [17]: academic training, professional performance, and scientific production. Selection of the content evaluators took place by means intentional sampling [18] through analysis of curricula on the Lattes Platform (simple search – search mode by subject matter) and consultation of the Groups and Research Directory (parameterized query – current base – search term), both from the National Council for Scientific and Technological Development (CNPq).

The semantic evaluation was carried out with 10 imprisoned women [19] enrolled in a state school located inside a female prison unit in the city of Recife, state of Pernambuco, northeastern Brazil. The inclusion criteria were as follows: women in closed or semi-open regimes and regularly enrolled in the chosen school. The women excluded were those that took part in the previously performed semantic evaluation stage corresponding to the data collection instrument called Knowledge about STIs.

### The Previna board game

The option for choosing the board game was based on the results of an integrative review^4^ and considering the particularities of the prison environment, in which the use of some educational technologies is restricted. Consequently, it was decided to choose an analog board game. The board game was developed based on an adaptation of the methodological framework developed by Aslan and Balci [20], called diGital educAtional gaMe dEvelopment methoDology (GAMED), which proposes the creation of digital educational games in 4 phases: game design, software design, game implementation and publication, and game-based learning and feedback. However, only the first phase was adopted in this research for dealing with a non-digital game.

For the purposes of selecting the content used in the game, the theoretical framework present in the Clinical Protocol and Therapeutic Guidelines for the Comprehensive Care of People with Sexually Transmitted Infections [21] was considered, as well as a query on the website of the Department of Chronic Conditions and Sexually Transmitted Infections belonging to the Brazilian Ministry of Health, diverse information for the general public, and the didactic content on STIs present in the biology book called Bio [22]. In addition to that, the Theory of Multiple Intelligences (TIM) guided the creation of the educational technology supporting the development of a board game focused on stimulating multiple intelligences [23, 24]. The main STIs associated with imprisoned women were considered, namely: HIV, Syphilis, Genital herpes, Viral hepatitis, Gonorrhea, Chlamydia and HPV [25–32].

The process to develop the game design underwent four stages: prototyping; game test, evaluation, and risk analysis [20]. The first three stages were used in this study, with the game test and evaluation phases corresponding to the content validation and semantic evaluation stages.

The board consisted of 27 squares presented in a lineal format with sinuosities. In addition to them, 1 square was devoted to the game Start and 1 for the end of the path. In an attempt to avoid monotony during the game, 5 squares were added to warn about risk behaviors or those targeted at STI prevention. When a player is in one these fields, she can move forward or backwards, according to the yellow card she takes from the corresponding stack. The other cards correspond to questions/challenges/information.

The game cards were developed with textual and graphic content on one side and answers at the bottom or in the alternatives themselves. The cards have different colors according to their type: true or false question (light blue); multiple-choice question (dark blue); challenge (purple); information (green); and prevention or risk (yellow). The game logo is on the back of each card.

Different types of questions were used for the game to be more appealing, with different difficulty levels. In addition to that, a type of card called “Information card” was created, which does not correspond to any question but to a reading activity that the players must perform. This strategy was used to ease learning of the content items considered as the most technical ones. Likewise, content was prepared to warn about STI risk behaviors or those for STI prevention.

The board game consisted of 01 board, 01 instruction manual, 05 pawns, 01 dice and 52 cards: 06 with true or false questions; 20 with multiple-choice questions; 05 with a challenge containing open questions; 07 with diverse information for reading; and 14 with information on prevention or risk.

### Content validation

After developing the game (version 1.0), it was submitted to the content validation process. In this stage, content validation was in charge of health and education professionals and graphic designers.

The professionals were emailed an invitation letter with the request to collaborate in the research. At the end of the text, a link was provided to access the necessary material for content validation of the Board Game: free and informed consent form; instrument to characterize the evaluators; instructions for content validation; and content validation instrument.

The Educational Health Content Validation instrument validated by Leite et al. [33] was used for the health and education professionals, with satisfactory overall internal consistency (ICC > 0.8). This instrument consists of 18 items distributed into three domains: objectives (purposes, goals or purposes); structure/presentation (organization, structure, strategy, coherence and sufficiency); and relevance (significance, impact, motivation and interest). The answer options are 0 (I disagree), 1 (I partially agree), and 2 (I totally agree).

For the game design/development area professionals, an instrument developed for face validation of educational technologies in health was used (CVI = 0.93). The instrument has 12 items, divided into 05 domains – objectives, organization, writing style, appearance and motivation – and has 05 answer options: I totally disagree, I disagree, I partially disagree, I agree, and I totally agree [34]. At the end, all the evaluators had the chance to make suggestions for the game, if they deemed it necessary.

### Semantic evaluation

After the adjustments made in the content validation stage, version 2.0 of the board game was prepared and the semantic evaluation process with the target population was initiated.

For data collection during the semantic evaluation, a verbal invitation was made to the imprisoned women enrolled at the prison school in the visit to the classroom under the supervision of the school director and the teacher responsible for the class. It was clear that participation was voluntary and anyone who was not interested in participating would do an activity in the classroom as part of school planning under the supervision of the school teacher. When they agreed to participate in the research, they were asked to sign the term informed consent. The document was read individually and any doubts were clarified. At a first moment, a characterization questionnaire was answered (social and demographic characteristics, prison situation, and sexual infections). The women were divided into 2 groups of 3 students and 1 group of 4 students. Subsequently, the game board and its rules were presented and a test game was conducted in the classroom. When the game ended, the women were invited to assess the game, through an individual interview, based on 15 questions divided into 3 categories: general aspects; playability; and design, adapted from the study by Taspinar, Schmidt and Schuhbauer [35].

### Statistical analysis

The final version of the database was transferred from Microsoft Excel® to the Stata software, version 15.0. Data analysis was initiated with sociodemographic and professional training and experience characterization, by calculating absolute and relative frequencies for the categorical variables, and mean, minimum, maximum and standard deviation for the numerical ones.

The answers given by the expert health and education professionals were analyzed from two perspectives: if the assessments were considered as numerical outcomes, mean calculation; and considering the assessments as categorical outcomes, by calculating absolute and relative frequency. Calculation of the Content Validity Coefficient (CVC) met the criteria set forth by Pasquali [36], in which the CVC of the items is calculated based on the mean values assigned by the experts/evaluators for each item, and subsequently divided by the maximum point of the Likert scale used, which is 2 in this study. In order to achieve the adjusted CVC, the Experts’ Polarization Error (EPE) was subtracted.

The answers given by the design experts were analyzed considering the assessments as numerical outcomes, by calculating the mean. In addition, the Positivity Index (PI) was calculated considering the number of positive answers in each dimension or item as numerator and the total number of answers as denominator. The assessments with grade 4 or 5 were considered positive. The CVC of the items was calculated based on the means assigned by the experts/evaluators for each of the items, being subsequently divided by the maximum point of the Likert scale used, which is 5 in this study. In order to achieve the adjusted CVC, the Experts’ Polarization Error (EPE) was subtracted. The cutoff point adopted to determine adequate content validity was ≥0.85 [36].

For the characterization of the incarcerated women, mean, minimum, maximum and standard deviation were calculated for the quantitative variables (age, number of children, years of study, income, current prison time and total prison time). Absolute and relative frequencies were used for the qualitative variables. Subsequently, absolute and relative frequencies were calculated for each item of the general aspects, playability and design components and the open questions about what they liked the most and suggestions for improvements analyzed in a qualitative way.

## Results

The process to validate the board game content involved 23 evaluators, with data analysis performed in two stages based on the use of different instruments for both groups. The first group consisted of 18 experts from the health (*n* = 13) and education (*n* = 5) areas; and the second group was comprised by 5 experts from the game design/development area.

As for the characterization of the health and education professionals, most of them were female (77.78%), aged between 32 and 71 years old with a mean of 48.27 (±10.96) and teachers (38.89%), followed by nurses (33.34%) with academic training at the PhD level (66.67%). Their training time varied from 5 to 46 years with a mean of 21.50 (±11.70), and their working time ranged from 4 to 46 years with a mean of 22.55 (±12.25). Most of the participants held the position of teacher (72.22%), and came from the Northeast region (61.12%).

In the process to validate the board game content, all 18 items evaluated obtained CVC values above 0.85. In addition to that, 4 items (1, 14, 15 and 18) obtained full agreement. The board game obtained a global CVC of 0.966 and, for that reason, it was considered valid in terms of content by this group (Table 1).

**Table 1:** Content Validation Coefficient for each item and set of items evaluated in the board game (Health and Education professionals)

Items			Evaluators’ assessment		
	Mean	CVC^a^	I disagree	I partially agree	I totally agree
			n (%)	n (%)	n (%)
1. It addresses the topic proposed	2.0	1.0	–	–	18 (100)
2. It is suitable to the teaching-learning process	1.94	0.972	–	1 (5.56)	17 (94.44)
3. It clears doubts about the topic addressed	1.88	0.944	–	2 (11.11)	16 (88.89)
4. It provides a reflection on the topic	1.94	0.972	–	1 (5.56)	17 (94.44)
5. It encourages a change in behavior	1.83	0.916	–	3 (16.67)	15 (83.33)
6. Adequate language for the target audience	1.77	0.888	–	4 (22.22)	14 (77.78)
7. Appropriate language for the educational material	1.94	0.972	–	1 (5.56)	17 (94.44)
8. Interactive language, allowing for active involvement in the educational process	1.94	0.972	–	1 (5.56)	17 (94.44)
9. Correct information	1.88	0.944	–	2 (11.11)	16 (88.89)
10. Objective information	1.94	0.972	–	1 (5.56)	17 (94.44)
11. Enlightening information	1.88	0.944	–	2 (11.11)	16 (88.89)
12. Necessary information	1.94	0.972	–	1 (5.56)	17 (94.44)
13. Logical sequence of ideas	1.94	0.972	–	1 (5.56)	17 (94.44)
14. Current topic	2.0	1.0	–	–	18 (100)
15. Adequate text size	2.0	1.0	–	–	18 (100)
16. It stimulates learning	1.94	0.972	–	1 (5.56)	17 (94.44)
17. It contributes to knowledge in the area	1.94	0.972	–	1 (5.56)	17 (94.44)
18. It arouses interest in the topic	2.0	1.0	–	–	18 (100)
**Set of items**	**1.93**	**0.966**	**–**	**22 (6.79)**	**302 (93.21)**

^a^Evaluators’ Polarization Error subtracted

In this stage, a total of 13 comments/suggestions were observed and, despite the CVC obtained, each comment/suggestion was individually evaluated in an attempt to refine the game. Grammatical corrections were made, the content was adjusted and 5 cards were added, their number thus rising from 47 to 52: 1 card about the preventive examination for cervical cancer - Prevention card: *“Have you undergone the preventive examination for cervical cancer”*; 3 cards about other prevention strategies foreseen in the HIV combined prevention mandala - Prevention card: *“You had unprotected sex but sought the health service and finished the treatment to prevent the HIV infection (Post-Exposure Prevention – PEP)”,* Prevention card: “*You used lubricating gel during sex to prevent the HIV infection”*, and Risk card: “*Your partner has HIV and you do not take the pill daily to prevent HIV infection (Pre-Exposure Prophylaxis – PrEP)*”; 01 T or F card about a doubt from a student in the classroom related to HIV transmission and mentioned by a prison school teacher – “*It is possible to transmit HIV through a cigarette butt*”.

As for the characterization of the game designers/developers, it was identified that 3 were female, aged between 28 and 46 years old and with a mean of 40.40 (±7.50) years, with training in the areas ofComputer Science (2), Information and Communication (1), Informatics in Education (1) and Networked Educational Technologies (1). Their training time varied from 7 to 25 years with a mean of 14.4 (±8.87) years, and their working time ranged from 7 to 24 years with a mean of 15.20 (±7.08) years. Most of them were from the South region of the country (3).

The content validation process related to the board game appearance involved an analysis of the illustrations, colors, and shapes and arrangement of the figures. In this stage, most of the items reached CVC values below 0.85. This is due to the small number of evaluators and to the metric being sensitive to differences in answers, as 01 negative answer was sufficient for the minimum index not to be obtained. The global value for the game was CVC = 0.750 and PI: 66.67% (Table 2).

**Table 2:** Content Validation Coefficient for each item and set of items evaluated in the board game – 1st round (Game designers/developers)

Items			Positive assessments		Negative assessments	
	Mean	CVC^a^	n	%	n	%
1. The illustrations are suitable for the target audience	3.6	0.720	4	80.0	1	20.0
2. The illustrations are clear and easy to understand	3.6	0.720	3	60.0	2	40.0
3. The illustrations are relevant for the target audience to understand the content	3.8	0.760	3	60.0	2	40.0
4. The colors of the illustrations are suitable for the type of material	3.6	0.720	3	60.0	2	40.0
5. The shapes of the illustrations are suitable for the type of material	3.6	0.720	3	60.0	2	40.0
6. The illustrations portray the everyday life of the target audience of the intervention	3.6	0.720	3	60.0	2	40.0
7. Arrangement of the figures is in harmony with the text	3.8	0.760	4	80.0	1	20.0
8. The figures used elucidate the content of the educational material	4.2	0.840	4	80.0	1	20.0
9. The illustrations assist in presenting the theme and follow a logical sequence	3.8	0.760	3	60.0	2	40.0
10. The number of illustrations included in the educational material is adequate	4.2	0.840	4	80.0	1	20.0
11. The size of the illustrations included in the educational material is adequate	3.8	0.760	4	80.0	1	20.0
12. The illustrations assist in changing the target audience’s behavior and attitudes	3.4	0.680	2	40.0	3	60.0
**Set of items**	**3.75**	**0.750**	**40**	**66.67**	**20**	**33.33**

^a^Evaluators’ Polarization Error subtracted

In the Comments and suggestions item, corrections were indicated regarding spelling, text font used, illustrations, logo, layout of the box and cards and graphics of the material, according to the excerpts below:

*For being a more serious part, the manual could have another font, not like sans, I suggest even a more common one like arial, calibri, titilium... (D1).*

*Another illustration that I think needs to be improved is the main one in the PREVINA game. The vast majority of the questions are asked in relation to the female companions; however, the illustration features a MALE X FEMALE pair. I think it would be good to do something more generic... I understand that one is a female and the other is a male condom, but I don’t know if it fits your target audience. Something to think about, actually. (D3).*

*The logo illustration, which is the most repeated and represents two condom models, is infantilized, transforming them into two anthropomorphic characters. This is inappropriate for the type of audience, which may even be offended by such infantilization. (D5).*

Based on these results and on the opportunities for improvement mentioned, it was decided to conduct a 2nd validation round with the game designers/developers. Five evaluators participated in this stage, four of whom were female and aged between 29 and 46 years old with a mean of 40.0 (±6.63), them being 2 teachers and 3 PhDs with training in the areas of Computer Science (2), Communication and Health Information (1), Intelligence and Design Technologies (1) and Networked Educational Technologies (1). Their training time varied from 2 to 20 years with a mean of 9.8 (±6.94), and their working time ranged from 7 to 23 years with a mean of 17.40 (±6.30). The Southeast region of the country was predominant (3), followed by the South region (2).

In the content validation process, it was noticed that all the items obtained CVC values ≥0.88 and the global value for the game was CVC = 0.917, with a positivity of 60, demonstrating that the changes made produced improvements in the new version of the game and allowed it to be considered validated (Table 3).

**Table 3:** Content Validation Coefficient for each item and set of items evaluated in the board game – 2nd round (Game designers/developers)

Items			Positive assessments	Negative assessments	
	Mean	CVC^a^	n	n	
1. The illustrations are suitable for the target audience	4.6	0.920	5	0	
2. The illustrations are clear and easy to understand	4.8	0.960	5	0	
3. The illustrations are relevant for the target audience to understand the content	4.6	0.920	5	0	
4. The colors of the illustrations are suitable for the type of material	4.8	0.960	5	0	
5. The shapes of the illustrations are suitable for the type of material	4.8	0.960	5	0	
6. The illustrations portray the everyday life of the target audience of the intervention	4.4	0.880	5	0	
7. Arrangement of the figures is in harmony with the text	4.4	0.880	5	0	
8. The figures used elucidate the content of the educational material	4.6	0.920	5	0	
9. The illustrations assist in presenting the theme and follow a logical sequence	4.6	0.920	5	0	
10. The number of illustrations included in the educational material is adequate	4.4	0.880	5	0	
11. The size of the illustrations included in the educational material is adequate	4.6	0.920	5	0	
12. The illustrations assist in changing the target audience’s behavior and attitudes	4.4	0.880	5	0	
**Set of items**	**4.58**	**0.917**	**60**	**0**	

^a^Evaluators’ Polarization Error subtracted

In the comments and suggestions some grammatical errors and surplus characters were indicated, as well as creation of new versions in Braille and digital as a way to make the technology more accessible to other audiences, in addition to praise for the evolution of the game in relation to the 1st version, as follows:

*The game idea and design are excellent. The rules are objective and easy to understand. It is obvious that you thought about the characters’ diversity, which is perfect. A suggestion into the future is to have a version with Braille. Another suggestion for further studies, and extrapolating the audience you were looking for at the moment, would be to transform it into a digital version, making such information available to other groups. (D3).*

*I would like to praise the researcher and all involved in the proposal of this game. The game is of good visual quality and the way in which the content is addressed eases its understanding. Another point that deserves to be highlighted is the evolution of the game, when comparing the 1st version with this one, it can be seen that there was progress and that some adjustments pointed out were improved. In addition to that, I would like to congratulate you on the topic chosen, as well as on the target audience, as it is essential to think about the diverse groups that comprise society. (D5).*

Based on the evaluators’ considerations, the material was revised again by the researcher and game development company, sent for Portuguese review and, after that, the prototype to be used in the semantic evaluation stage was made.

The participants of the semantic evaluation were 10 women aged between 21 and 35 years old with a mean of 28 (±5.24), single (9), mixed race (8), and mostly from Pernambuco (9) with Recife standing out (6). The number of years of study varied from 5 to 15 with a mean of 9.05 (±2.78), and family income ranged from R$ 99.00 to R$ 7000.00 with a mean of R$ 1761.33 (±2090.66). In terms of children, 7 said they had, and the number varied from 1 to 5, with a mean of 2.1 (±1.79). Most of them had been arrested for the first time (6), with current prison time between 4 and 29 months, mean of 10.9 (±7.95) months and total prison time between 17 and 120 months, with a mean of 81.0 (±44.60) months. The majority (7) was awaiting trial.

With regard to STIs, 8 stated that they had already received diverse information on the subject matter from health professionals (6), in health centers (3) and via the Internet (2). Referring to doubts related to the STIs, only 3 stated having some, especially about syphilis, discharges, myths and truths about STIs. These doubts were normally resolved with health professionals (5) and family members (3). Regarding history of STIs, 2 stated having had syphilis, and 1 mentioned that her partner also had STIs, although she did not know which. In terms of treatment, 2 women mentioned that only them underwent it.

In the evaluation of the game, on general aspects, the women indicated that they liked the cards and questions, all the information, the content, the explanations, the possibility of discussing and solving doubts, the characters, the interaction and the group dynamics. In terms of suggestions, only the possibility of using recyclable material to build it was mentioned. In addition to that, all the women indicated that they improved their knowledge, increased their motivation to attend the class, and that they would like to play the board game again. This can be observed in the statements below referring to the item about the aspect they liked the most in the game:

*The True and False questions, which make the game interesting. (E1).*

*About the information on STIs, questions and clarifications. (E2).*

*The questions, the information, the cards. (E3).*

*It draws the attention of people that are not aware of STIs and the game explains each of them very well. (E4).*

*The game, the questions and, as it is a group game, it allows discussions and, therefore, solving doubts. (E5).*

*I liked the content, the cards, the characters and the interaction. (E6).*

*The questions and the group dynamics. (E7).*

*Varied questions; content; new knowledge. (E8).*

*I liked the game; learning new knowledge. (E9).*

*About the information... the game allowed solving doubts. (E10).*

Likewise, in terms of playability, all of them considered the rules clear, had no problems understanding the game’s instructions, considered the questions’ level adequate and had fun playing it. The game lasted 45 minutes.

**Fig. 1:**
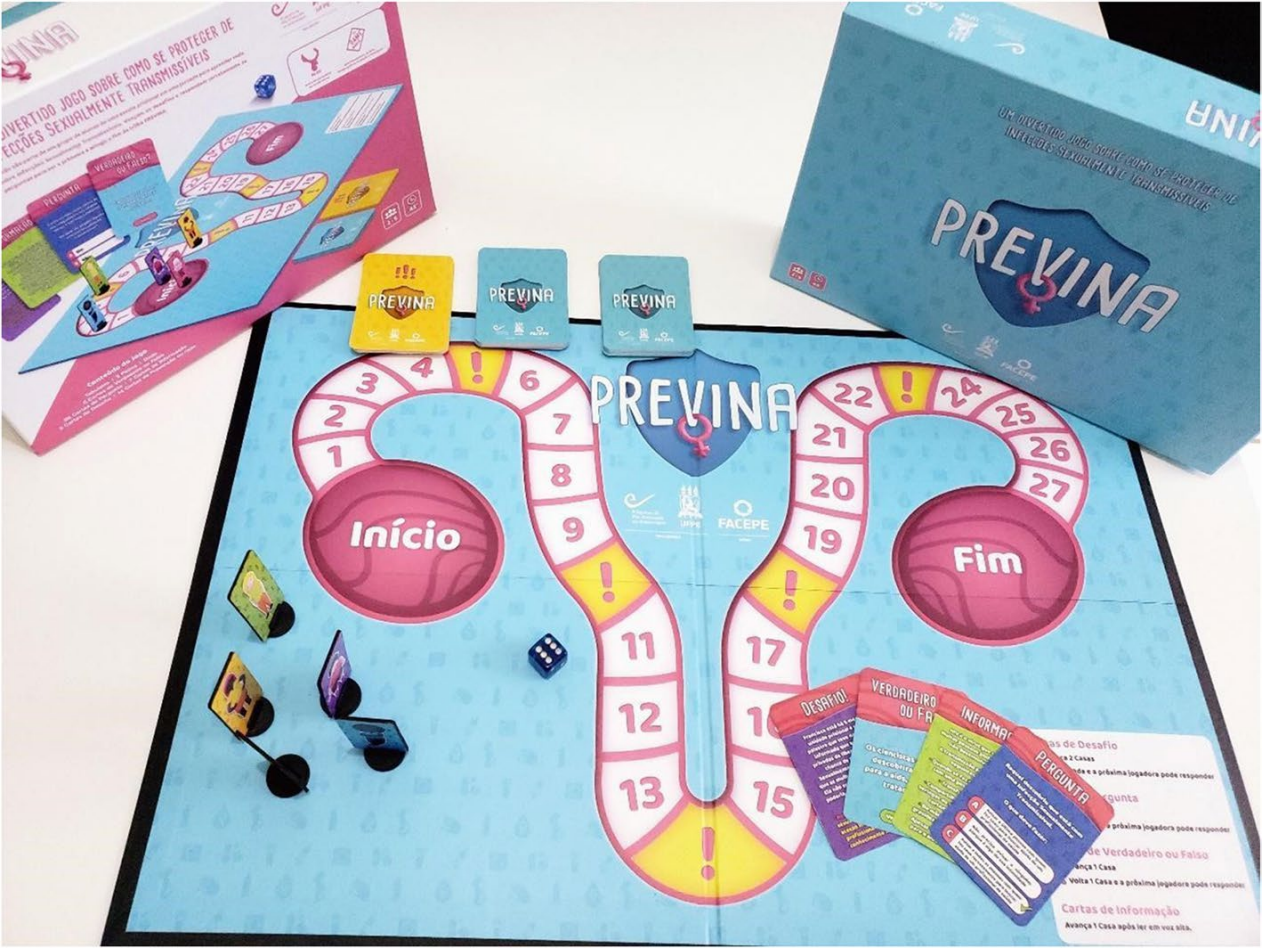
Final version of the Previna board game

Regarding the design, all the women considered the drawings appealing and the number of board squares and questions as adequate. They also indicated that they liked the question cards and the several types of questions increased their motivation. Figure 1 presents the final version of the game.

## Discussion

The Previna board game was based on a collective construction with contributions from health professionals, prison school teachers, game designers/developers and imprisoned women. The result is an educational technology that does not intend to exhaust all content on STIs, but to provoke reflections on a subject matter that is difficult to address in the school environment, especially in the prison context.

The game was developed taking into account the reality of the context experienced by imprisoned women, which may allow more significant learning. This type of educational technology can be used in different knowledge fields, being an option to be considered in the educational improvement process, stimulating involvement and motivation [15].

For its development, it was essential to adopt an appropriate theoretical and methodological framework that significantly impacted on the quality of the educational technology developed. The TMI broke with the traditional intelligence concept and enabled a new universe of possibilities for educators who, through this theory, have subsidies to develop a more personalized education based on respect for the differences between students and their ways of learning [37]. Thus, the board game developed was designed with components capable of stimulating different intelligences: linguistic, logical-mathematical, interpersonal, intrapersonal and spatial. In addition to that, it provides a learning environment that involves the player and holds her attention, due to situations that reflect the experiences of women in the prison context.

In turn, the methodological framework proposed by Aslan and Balci^20^ for the development of educational games allowed summarizing the best strategies to design the board game. This framework has also been used to design games in the education context [38, 39].

The content validation carried out by different health and education professionals, the latter members of the prison school where the study was developed, allowed developing a game guided by the view of different knowledge areas and aligned with the particularities of the target population. The participation of different professionals in the analysis of the board game as an educational technology is a common practice in validation processes [40–42]. These processes allow refining the material produced from the perspective of multidisciplinarity.

In this process of adjustments, the health professionals suggested the inclusion of new content in the cards and other STI prevention strategies recommended by the Brazilian Ministry of Health, namely: PrEP, PEP, use of lubricating gel associated with condoms, and performing the preventive examination for cervical cancer [21]. This confers emphasis to the central idea of the game, prevention, a concept that is presented in its very name: Previna (Prevent in Portuguese). In addition to that, it reinforces a combination of actions that these women can follow focused on risk reduction and on safer sex practices within their specific realities.

In addition, including cards with content about PrEP and PEP and raising discussions about them can help reduce the knowledge gap about these prevention strategies in the Brazilian population [43]. Likewise, the presence of content on cervical cancer and its association with STIs is a way of informing about the subject matter with a focus on recognizing risks and assuming responsibility for their own sexual health [44].

The assessment made by the teachers of a prison school in relation to the material was very positive, and the suggestion to include a card arose from the experience of one of them. He had to deal with a doubt from a student about the possibility of HIV transmission through the saliva present in shared cigarettes, a common practice among imprisoned women in that environment. Regarding the subject matter, it has already been evidenced that saliva breaks HIV particles in vitro and that many substances found in saliva inhibit and inactivate HIV particles [45]. A systematic review evidenced that there is no HIV transmission risk in the act of spitting and that, even in cases of bites, the risk is insignificant [46]. This reinforces the role of the game to break with taboos and clarify doubts inherent to the prison setting.

The participation of professional designers allowed improving the material aspect in order to make it more appealing and aligned with the characteristics of the imprisoned women. During the development process, it was sought to ensure that the women felt represented by the board game. This is reflected in details such as the board design itself in the shape of the female reproductive system, the symbol of the feminine in the name Previna and the presence of pawns with different profiles of women.

A game with an appealing plot, adapted to the gender and age group, and with characters with which the players can identify, are resources capable of increasing user engagement [47]. Thus, when present in the game, certain elements can increase the players’ motivation and engagement during learning, being able not only to improve knowledge acquisition, but also to promote attitudinal changes [48].

As a result of a rigorous content development and validation process and of the adjustments made, previously, in the semantic evaluation stage, the women positively evaluated the educational technology both in its general aspects and in terms of playability and design and did not propose any suggestions for changes, except for considering the use of recyclable material in a future printed version. In addition to having fun while playing, it was evident that they identified themselves with the material.

This reinforces the concept that the development of a participatory educational technology with the presence of different actors of society involved in the study object can contribute to the construction of knowledge and skills between academia and the community [40]. Although knowledge acquisition has not yet been tested, as a result of using the board game, it is possible that it may be able to sensitize women towards issues such as STI transmission, treatment and prevention strategies.

Among the main limitations is the impossibility of using Internet-associated materials for the game to be more interactive. In addition to that, although it is possible to play the game without the presence of a mediator, represented by a teacher, health professional or even a re-educator with more knowledge on the subject matter, it is recognized that their participation can make the discussion more productive. Therefore, it is suggested to incorporate educational technology during prison classes, in any of the shifts, morning, afternoon or night, or in health education activities carried out in the school or prison unit. The game can be used as part of broad interventions that involve self-care actions, STI control, and development of skills and practices that promote sexual and reproductive health.

Finally, for an individual game to be played, it is important that the women are motivated to learn by means of this methodology and they already have some knowledge about STIs.

## Conclusions

The Previna board game, validated by judges and evaluated by the target audience, presents itself as an important pedagogical tool in the construction of knowledge about STI prevention, treatment and control in the context of a female prison environment. The quality of this educational technology is directly related to its development based on an appropriate theoretical and methodological framework, in addition to the feedback from the target audience that was involved with the game from the association between reflection and fun, which constituted a stage for learning. It is recommended that the effect of the board game in question be evaluated through quasi-experimental or experimental studies, regarding the level of knowledge about STIs of imprisoned women.

## Data Availability

The data generated and used in the analysis of this study are included in this article. The authors will provide additional data upon reasonable requests.

## Abbreviations

STI: (Sexually Transmissible Infection)
HIV/AIDS: (Human Immunodeficiency Virus/Acquired Immunodeficiency Syndrome)
CNPq: (*Conselho Nacional de Desenvolvimento Científico e Tecnológico*)
TMI: (Theory of Multiple Intelligences)
GAMED: (diGital educAtional gaMe dEvelopment methoDology)
CVI: (Content Validity Index)
ICC: (Intra-Class Coefficient)
CVC: (Content Validity Coefficient)
EPE: (Experts’ Polarization Error)
PrEP: (Pre-Exposure Prophylaxis)
PEP: (Post-Exposure Prophylaxis)
